# Intravital Observation of Plasmodium berghei Sporozoite Infection of the Liver

**DOI:** 10.1371/journal.pbio.0030192

**Published:** 2005-05-24

**Authors:** Ute Frevert, Sabine Engelmann, Sergine Zougbédé, Jörg Stange, Bruce Ng, Kai Matuschewski, Leonard Liebes, Herman Yee

**Affiliations:** **1**Department of Medical and Molecular Parasitology, New York University School of MedicineNew York, New YorkUnited States of America; **2**Department of Parasitology, Heidelberg University School of MedicineHeidelbergGermany; **3**Department of Medical Oncology, New York University School of MedicineNew York, New YorkUnited States of America; **4**Department of Pathology, New York University School of MedicineNew York, New YorkUnited States of America; Medical Biotechnology LabsUganda

## Abstract

*Plasmodium* sporozoite invasion of liver cells has been an extremely elusive event to study. In the prevailing model, sporozoites enter the liver by passing through Kupffer cells, but this model was based solely on incidental observations in fixed specimens and on biochemical and physiological data. To obtain direct information on the dynamics of sporozoite infection of the liver, we infected live mice with red or green fluorescent Plasmodium berghei sporozoites and monitored their behavior using intravital microscopy. Digital recordings show that sporozoites entering a liver lobule abruptly adhere to the sinusoidal cell layer, suggesting a high-affinity interaction. They glide along the sinusoid, with or against the bloodstream, to a Kupffer cell, and, by slowly pushing through a constriction, traverse across the space of Disse. Once inside the liver parenchyma, sporozoites move rapidly for many minutes, traversing several hepatocytes, until ultimately settling within a final one. Migration damage to hepatocytes was confirmed in liver sections, revealing clusters of necrotic hepatocytes adjacent to structurally intact, sporozoite-infected hepatocytes, and by elevated serum alanine aminotransferase activity. In summary, malaria sporozoites bind tightly to the sinusoidal cell layer, cross Kupffer cells, and leave behind a trail of dead hepatocytes when migrating to their final destination in the liver.

## Abstract 2

Tiny parasites of the Plasmodium genus cause malaria. New details of the parasites' life cycle are uncovered through the use of intravital microscopy to observe the parasites' infiltration of the liver.

## Introduction

**Figure 1:**
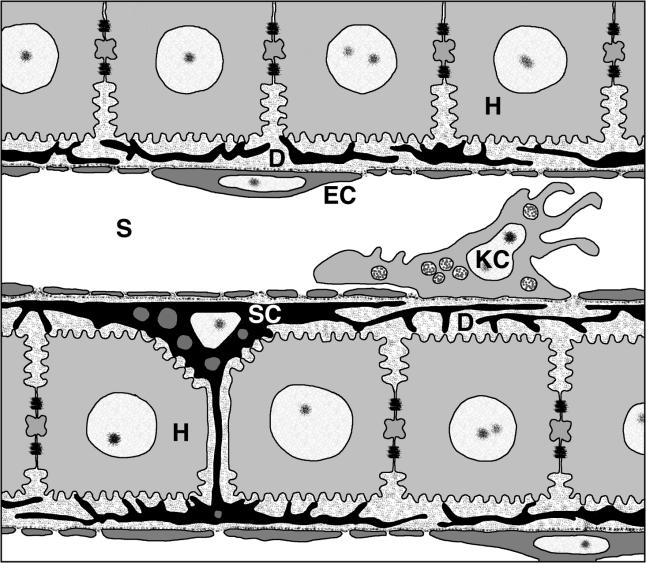
Architecture of the Liver Sinusoid Liver sinusoids (S) are lined by fenestrated endothelia (EC) and interspersed Kupffer cells (KC), the resident macrophages of the liver. Stellate cells (SC), the major producers of liver ECM, are located inside the narrow space of Disse (D), which is formed by the sinusoidal cell layer and cords of hepatocytes (H).

After transmission by an infected mosquito, malaria sporozoites enter a blood vessel at the bite site [1] and travel via the bloodstream to the liver, their initial site of replication in the mammalian host. But to gain access to hepatocytes, where they replicate, sporozoites must first recognize and arrest in the liver and then cross the sinusoidal cell layer, which is composed of specialized, highly fenestrated endothelia and Kupffer cells, the resident macrophages of the liver (Figure 1).

Initial data had indicated that arrest in the liver is mediated by binding of *Plasmodium* sporozoites via their major surface proteins, circumsporozoite protein (CSP) and thrombospondin-related adhesive protein, to the unique heparin-like oligosaccharides in hepatic heparan sulfate proteoglycans [2–10]. Analysis of the individual glycosaminoglycans produced by the major liver cell types revealed that stellate cells, perisinusoidal fat-storing cells of a dendritic shape [11], synthesize eight times more sulfated proteoglycans than hepatocytes and secrete most of these proteoglycans into the extracellular matrix (ECM) [12,13]. Recent work showed that CSP interacts with large secreted proteoglycans from stellate cells, distinct chondroitin and heparan sulfate proteoglycans on the surface of Kupffer cells, and heparan sulfate proteoglycans expressed on the surface of hepatocytes, but not with proteoglycans synthesized by sinusoidal endothelia [4]. Our previously published data indicate that the initial arrest of malaria sporozoites in the liver is mediated by the large stellate-cell-derived ECM proteoglycans that protrude from the space of Disse through the endothelial sieve plates into the sinusoidal lumen [4,14]. The parasites then glide along the endothelial cell layer until they recognize proteoglycans expressed on the surface of Kupffer cells. The best evidence obtained from fixed specimens and biochemical and physiological data suggests that sporozoites enter the liver by passing through Kupffer cells [4,14–21], although it has been suggested they cross directly through the fenestration of sinusoidal endothelia [22]. Support for Kupffer cells being the portal to the liver parenchyma has come from an in vitro model based on purified P. berghei and P. yoelii salivary gland sporozoites and Kupffer cells isolated from rat livers [14] showing that sporozoites actively invade Kupffer cells, enter a nonfusogenic vacuole, and exit the phagocytes unharmed. Further support for Kupffer cells being the gate to the liver derives from mutant P. berghei strains deficient in either SPECT1, a micronemal protein of unknown function, or SPECT2, a sporozoite protein with a putative membrane attack complex domain, which exhibit greatly diminished infectivity [23,24]. However, the mechanism of action of these proteins, their role in sporozoite entry into the liver, and the fate of the mutant sporozoites in the infected host remain to be established. Our hypothesis is that sporozoites actively enter Kupffer cells, traverse them by forming a nonfusogenic vacuole, exit them unharmed toward the space of Disse [4,14,16,17], and then migrate through several hepatocytes before eventually settling down in a final one for multiplication and differentiation to thousands of merozoites [25].

As stated above, the current model is based on a collection of indirect data. Direct confirmation of this model is essential prior to further development of our understanding of the molecular events involved in this process. However, direct, real-time observation of malaria sporozoite invasion within the liver is difficult, because the liver is a dense internal organ and the chance of observing a sporozoite in a limited viewing range requires inoculation of large numbers of sporozoites. Although large numbers of sporozoites can be routinely isolated from mosquito salivary glands, only a minority of these is infective in vitro. Even with optimized conditions, P. berghei invasion rates reach only 10%–20% in vitro [26], perhaps because a small percentage of the sporozoites in mosquito salivary glands are infectious [27,28]. Here, we present an approach that has overcome these obstacles through use of intravital microscopy for direct observation of fluorescent P. berghei sporozoites transmitted naturally by the bite of infected Anopheles stephensi mosquitoes. We were able to directly observe within the livers of live mice and rats the process of sporozoites being arrested within liver sinusoids, migrating along the sinusoid, entering and passing through Kupffer cells, traversing and damaging hepatocytes, and, finally, entering and remaining within hepatocytes.

## Results

### Intravital Studies

**Figure 2:**
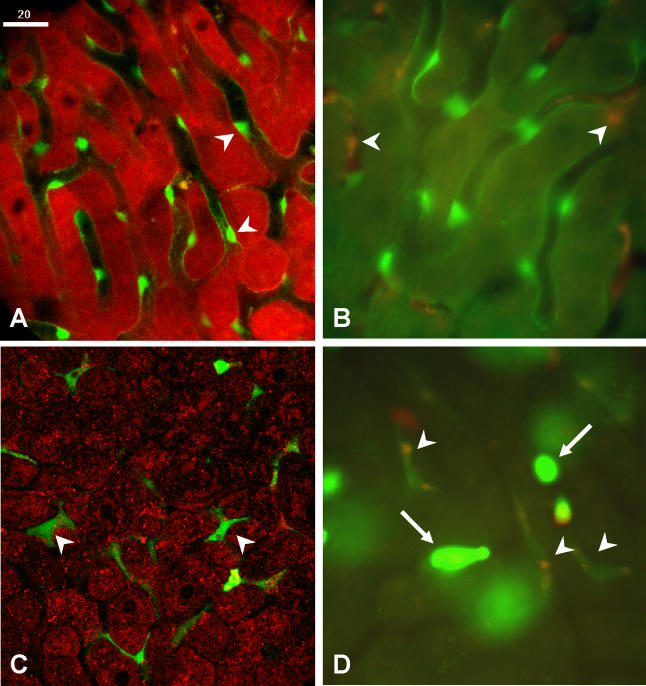
Distribution of GFP-Expressing Endothelia and Kupffer Cells in the Liver (A) Confocal microscopy demonstrates the GFP distribution in sinusoidal endothelia from a Tie2-GFP mouse. GFP is most prominent in the perinuclear region (arrowheads) of endothelia located in the periphery of the liver lobule. (B) A still image from an intravital movie shows GFP-expressing endothelia lining the sinusoids of a Tie2-GFP mouse. Kupffer cells can be identified by their orange autofluorescent lysosomes (arrowheads). (C) Star-shaped Kupffer cells (arrowheads) are located in sinusoids of a lys-EGFP-ki mouse liver. (D) Round blood granulocytes (arrows), traveling with the bloodstream or crawling along the sinusoidal cell layer, exhibit a stronger GFP signal than Kupffer cells (still image extracted from an intravital movie). Note the orange autofluorescence of the Kupffer cell lysosomes (arrowheads). Bar = 10 μm. See Videos S1 and S2.

Mice or rats were placed ventral side down on the stage of an inverted digital microscope with the liver exposed and immobilized so as to limit to a minimum motion caused by breathing. Typically, this allowed a 0.5–1-cm^2^ area of the liver surface to be examined by intravital microscopy. We were aided by the use of transgenic Tie2-GFP and lys-EGFP-ki mice, which express green fluorescent protein (GFP) in endothelia and in phagocytes (including Kupffer cells), respectively [29,30], thus allowing us to identify sinusoidal cell types in the liver. GFP was excited at 488 nm and documented by confocal or digital epifluorescence microscopy. The natural autofluorescence emission of the tissue allowed simultaneous revelation of the liver architecture. Confocal laser scanning as well as conventional epifluorescence microscopy permitted viewing through the intact liver capsule into the parenchyma to a depth of up to 50 μm so that we could observe the entire peripheral layer of hepatocytes and sinusoidal capillaries. In the Tie2-GFP mice, GFP was expressed predominantly by endothelia of the periportal zone of the liver lobule (Video S1; Figure 2A and 2B), while in the lys-EGFP-ki mice, Kupffer cells throughout the entire lobule exhibited equal GFP signals (Video S2; Figure 2C and 2D). Neutrophil granulocytes could be distinguished by their shape and by a GFP signal brighter than that of Kupffer cells.

Sporozoite infection for intravital examination was done exclusively by mosquito bite for the following reasons. First, the continuing biting activity of the mosquitoes and the gradual escape of the parasites from the skin [1,22,31] allowed examination of sporozoites entering the liver over a period of at least 3 h. Intravenous sporozoite injection would have narrowed the window of analysis to a few minutes [22]. Second, electron microscopic examination revealed that routine sporozoite preparations are highly contaminated with salivary gland debris (U. Frevert, unpublished data). Intravenous inoculation would have caused most of this mosquito debris to be cleared from the bloodstream by Kupffer cells. The consequences of extensive phagocytosis of foreign material for sporozoite passage would have been unpredictable. Third, in agreement with the notion that only a small pool of the *Plasmodium* sporozoites in salivary glands may be infectious [27], the vast majority of purified salivary gland sporozoites are unable to infect hepatoma cells in vitro, even under optimal conditions [26]. Inoculation of a mixture of infectious and noninfectious parasites would have obscured the difference between active sporozoite invasion and phagocytosis of dead or damaged parasites. We reasoned that sporozoites that are able to leave the skin at the mosquito bite site [1] compose in all likelihood the most viable parasite pool and therefore reflect the onset of the liver phase of the malaria life cycle most accurately.

**Figure 3:**
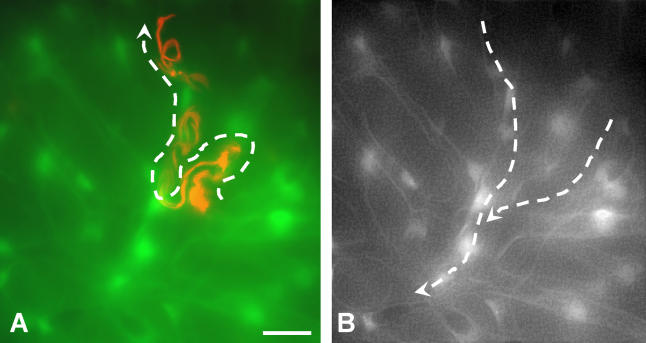
Sporozoite Gliding along the Sinusoidal Endothelium (A) A P. berghei sporozoite expressing fluorescent RedStar protein glides with and against the bloodstream inside a liver sinusoid of a Tie2-GFP mouse. The arrow indicates the overall movement of the parasite. (B) The projection through the same area of the liver visualizes the outline of the highly branched sinusoids. The direction of the blood flow is indicated by the dashed arrows. Bar = 10 μm. See Videos S3 and S4.

**Figure 4:**
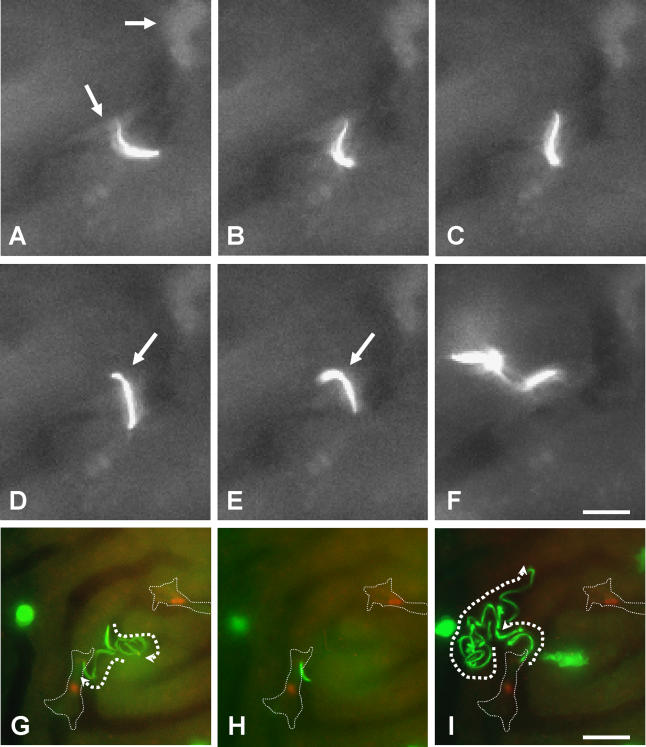
Sporozoite Passage into the Liver Parenchyma (A–E) show individual frames extracted from an intravital movie; (F) is a projection visualizing the transmigration path of the GFP P. berghei sporozoite in a Tie2-GFP mouse liver. (G–I) show projections from an intravital movie demonstrating the path of the parasite; its overall direction is indicated by arrows (dotted lines). (A) After gliding along a sinusoid, a sporozoite has encountered a Kupffer cell, which it faces with its apical cell pole. (B and C) Following a pause, the parasite slowly enters the Kupffer cell. (D and E) Sporozoite passage into the liver parenchyma occurs at a slow speed and involves the formation of a constriction in the parasite (arrow). (F) Once inside the liver tissue, the sporozoite increases its speed and transmigrates through several hepatocytes. (G) A projection from an intravital movie shows the path of a GFP P. berghei sporozoite gliding against the bloodstream along a sinusoid in a lys-EGFP-ki mouse liver. Eventually, the parasite encounters a Kupffer cell. (H) The sporozoite stops, facing the phagocyte with its apical cell pole. The outline of the two Kupffer cells in the image is indicated by dotted lines. (I) After slowly passing through the Kupffer cell, the sporozoite enters the liver parenchyma and migrates through several hepatocytes. Bars = 10 μm. See Videos S5 and S6.

**Figure 5:**
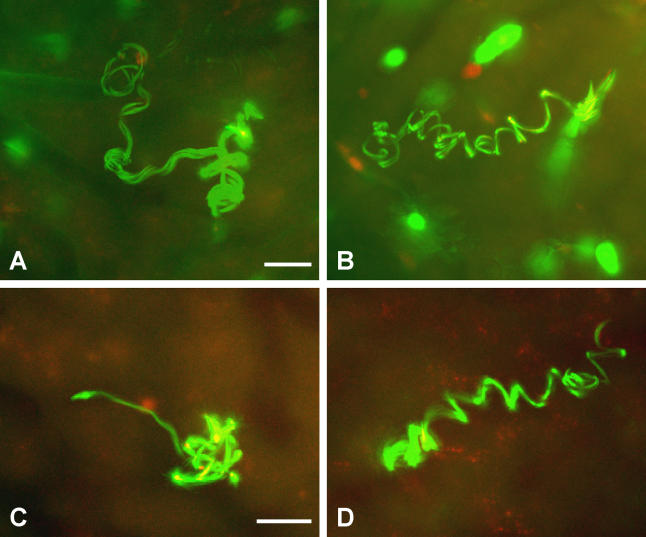
P. berghei Sporozoite Transmigration Is Independent of the Species of the Infected Host Projections of typical GFP P. berghei sporozoite paths show that the parasites transmigrate in a similar fashion through many hepatocytes in mouse ([A] Tie2-GFP mouse; [B] lys-EGFP-ki mouse) and also in rat livers (C and D). Bars = 10 μm. See Videos S7 and S8.

**Figure 6:**
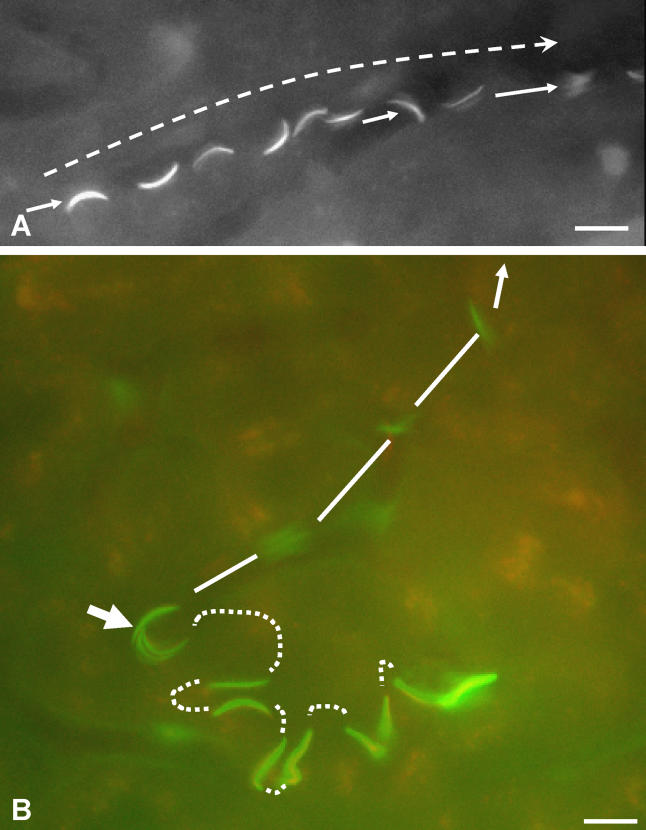
Unsuccessful Attempts of Liver Infection (A) The composite image of ten selected frames from an intravital movie shows a paralyzed or dead P. berghei sporozoite that is eventually dislodged and flushed out of the liver lobule (short arrows). The parasite maintains a fixed crescent shape, fails to cling to the sinusoidal cell layer, and makes no attempt to glide against the bloodstream. The long arrow (dotted line) indicates the direction of the blood flow. (B) Projection composed of 14 selected frames extracted from an intravital movie showing a GFP P. berghei sporozoite that initially transmigrates in the liver parenchyma (dotted lines), but then reenters a sinusoid (arrow) and is swept away with the bloodstream (solid lines). Bars = 10 μm. See Videos S9 and S10.

In the 26 mice examined, a total of 86 P. berghei sporozoites were analyzed, ranging from one to 14 parasites per mouse. P. berghei sporozoites were detected at various stages of the liver infection cascade. The dynamic aspects of the events are critical to the proper interpretation of the data (see Videos S1–S13). Eleven (12.8%) sporozoites were observed while they were gliding along the sinusoidal cell layer (Videos S3 and S4; Figure 3A and 3B) and then crossing it. Four of these observations were made in lys-EGFP-ki mice, and in all four cases, passage occurred through a Kupffer cell (Videos S5 and S6; Figure 4). Another seven sporozoites were found crossing this barrier in Tie2-GFP or Balb/c mice or in Brown Norway rats in a similar fashion (data not shown). In these animals, Kupffer cells were identified by their orange autofluorescent lysosomes (see, e.g., Figure 2D). Forty-six (53.5%) sporozoites had already entered the liver tissue at the time of detection and were in the process of transmigrating through hepatocytes (Videos S7 and S8; Figure 5). Ten (11.6%) sporozoites were sitting still inside a hepatocyte and had apparently completed the transmigration phase. Fifteen (17.4%) parasites were caught in the process of gliding along the sinusoidal cell layer but failed to pass into the liver tissue (Video S9; Figure 6A), and four (4.7%) sporozoites initially migrated in the tissue, but then re-entered the bloodstream and were flushed out of the liver lobule (Video S10; Figure 6B). The maximum time of sporozoite migration observed was approximately 15 min, and the longest covered distance was an estimated 400–500 μm (i.e., 40 to 50 times the length of a sporozoite).

When being transported passively with the bloodstream, sporozoites travel at a speed of 11.2 ± 5.7 μm/s (Table 1). For comparison, the sinusoidal velocity of neutrophil blood granulocytes in lys-EGFP-ki mice was 29.0 ± 13.9 μm/s. The analysis was restricted to phagocytes traveling free in the bloodstream; cells crawling along the sinusoidal cell layer were excluded. We observed sporozoites entering a liver lobule to be abruptly arrested by binding to the sinusoidal cell layer (data not shown). After a short pause, these sporozoites began gliding along the sinusoidal cell layer at a speed of 1.7 ± 0.7 μm/s (Table 1). Neither the shear force of the blood plasma nor the repeated encounters with passing blood cells released attached parasites from the sinusoidal cell wall, suggesting adhesion with high affinity. This conclusion is supported by our observation that sporozoites glided on sinusoidal cell layer equally well with and against the direction of blood flow. Paralyzed or dead parasites were characterized by a fixed crescent shape and no movement against the bloodstream (see Video S9; Figure 6A). They seemed not to adhere as well, since they were frequently released from the sinusoidal cell layer and eventually flushed out of the liver lobule.

**Table 1:**
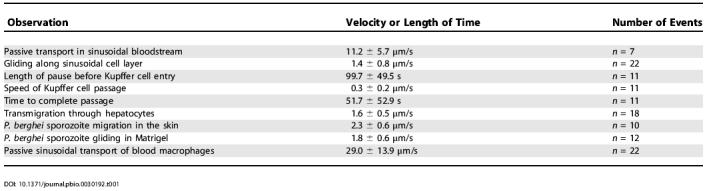
*Plasmodium* Sporozoite Migration Speed

Prior to crossing the sinusoidal cell layer, sporozoites adhered with their apical end to a Kupffer cell. After a pause of 99.7 ± 49.5 s (see Videos S5 and S7; Figure 4A–4I; Table 1), the parasites slowly pushed, at a speed of 0.3 ± 0.2 μm/s, through a clearly visible constriction (see Video S5; Figure 4D and 4E). The time required for completing the passage was 51.7 ± 52.9 s. Compared with the velocities of sporozoite gliding along the sinusoidal cell surface and sporozoite transmigration through hepatocytes, which did not differ significantly from each other or from sporozoite gliding in Matrigel (Video S11; Table 1), the sporozoite passage through Kupffer cells was slower by an order of magnitude (*p* ≤ 0.0001). Since the gliding speed in Matrigel (1.8 ± 0.6 μm/s) is similar to the speed within the liver and since the migration pattern in Matrigel resembles that in skin and liver, we think that gliding in Matrigel offers a reasonable model for the study of sporozoite gliding.

Once inside the liver parenchyma, sporozoites increased their speed to 1.6 ± 0.5 μm/s (Table 1), seeming to effortlessly breach the plasma and intracellular membranes of several hepatocytes (see Video S6; Figure 4I) and most likely causing substantial damage to the subcellular architecture (Video S12). Sporozoites were observed transmigrating through the liver parenchyma for up to 15 min before eventually settling down in a final hepatocyte. Sporozoite transmigration through hepatocytes differed clearly from Kupffer cell passage in that this mode of invasion did not involve the formation of a constriction, a pause before entry, or a decrease in migration speed. In contrast to in vitro gliding, the parasites were highly flexible in vivo and frequently changed their direction. In some cases, sporozoites passed across tissue barriers in a manner that cannot be explained solely by gliding motility: parasites were observed to compress or coil up before crossing the barrier and to use their posterior end to propel themselves across the obstacle (Video S13).

To document the path of migrating sporozoites, projections were generated from digital movies. The parasites generally followed the architecture of the liver lobule, i.e., the sinusoidal lumen (see Videos S3, S6, S9, and S10; Figures 3A, 4G, 6A, and 6B) or the hepatocyte chords (see Videos S6, S7, S8, and S10; Figures 4I, 5A–5D, and 6B). Overall, 77.9% of the P. berghei sporozoites traveling in the sinusoidal blood were able to enter the liver parenchyma of mice—which is not to say, however, that all these sporozoites would have matured to exoerythrocytic forms (EEFs). A few sporozoites were observed re-entering the bloodstream after successful transmigration in the liver parenchyma and being flushed out of the liver lobule (see Video S10; Figure 6B).

Compared with Balb/c mice, young Brown Norway rats are considerably more susceptible to infection with P. berghei sporozoites, coinciding with a markedly less pronounced inflammatory response [32]. Since the inflammatory response of the host is thought to eliminate a large proportion of the EEFs in mice, we reasoned that a shorter transmigration phase, resulting in a lower number of dead hepatocytes, would explain the higher sensitivity of rats to P. berghei infection. To test this hypothesis, we infected young Brown Norway rats with green fluorescent P. berghei sporozoites. Surprisingly, intravital examination of the livers of these animals demonstrated the same general sporozoite migration behavior as in mice (see Videos S7 and S8; Figure 5A–5D), suggesting that the extent of hepatocyte transmigration does not directly correlate with the degree of hepatocyte death and/or inflammation.

### Histopathology

**Figure 7:**
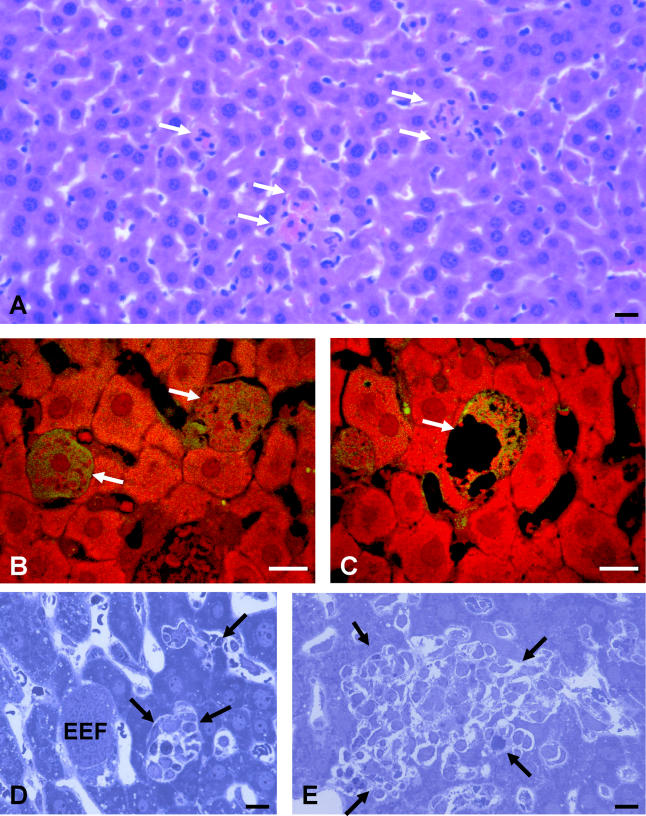
Transmigrating *Plasmodium* Sporozoites Leave Behind a Trail of Dead Hepatocytes (A) Three hours after infection with 5 × 10^6^ P. berghei salivary gland sporozoites, a mouse liver contains small clusters of necrotic hepatocytes that have been infiltrated by inflammatory cells (arrows). (B) Four hours after intravenous infection with 5 × 10^6^ P. yoelii sporozoites, a mouse liver contains individual or small clusters of necrotic hepatocytes (arrows). (C) Six hours after infection, the signs of hepatocytic damage appear more severe in another mouse liver (arrow). (D) Forty hours after inoculation of 2 × 10^6^ P. yoelii sporozoites, small infiltrates of inflammatory cells (arrows) block the lumina of some sinusoids, while maturing EEFs are free of any inflammatory reaction. (E) Fifty hours after infection with 2 × 10^6^ P. yoelii sporozoites, the size of the inflammatory infiltrates (arrows) has increased. Stains used: (A) paraffin section stained with H&E, (B and C) frozen sections stained with Evans blue, (D and E) semithin Epon sections stained with Toluidine blue. Bars = 20 μm.

**Figure 8:**
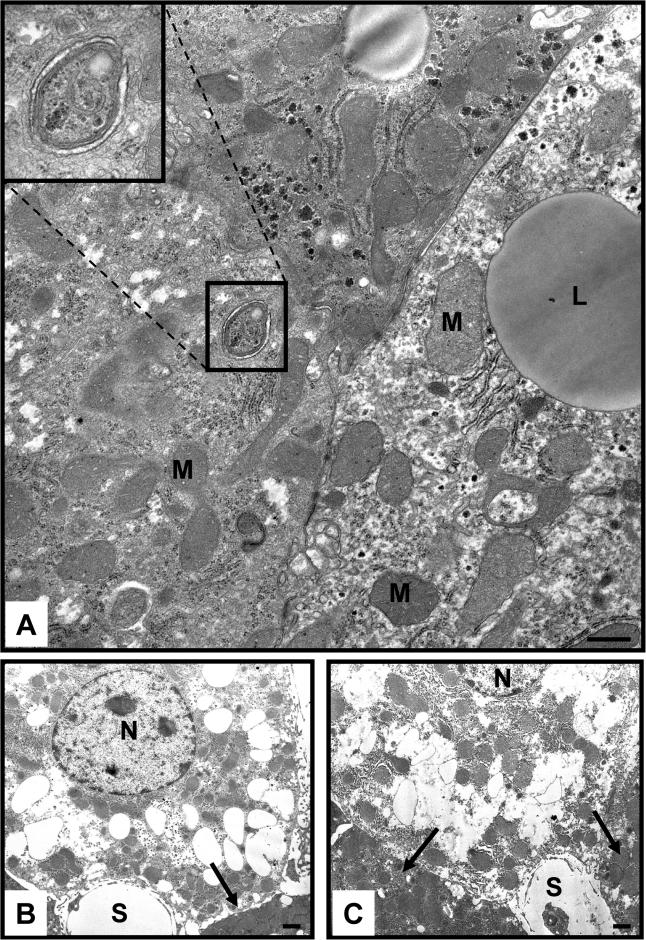
Sporozoites, Surrounded by a Parasitophorous Vacuole Membrane, Can Be Found in Intact Hepatocytes (A) Six hours after infection by bite of 50 P. yoelii–infected mosquitoes, electron microscopic examination of a mouse liver shows a sporozoite inside an intact hepatocyte. Note that the parasite is enclosed in a parasitophorous vacuole (insert). The neighboring hepatocyte shows signs of cytoplasmic swelling. (B and C) P. yoelii–infected mouse livers contain hepatocytes that exhibit various degrees of necrosis, ranging from hydropic swelling to near-complete disintegration of the cell, adjacent to parenchymal cells with a normal ultrastructure (arrows). The normal hepatocytes did not contain a sporozoite in the plane of the section. L, lipid droplet; M, mitochondrium; N, nucleus; S, sinusoid. Bars = 1 μm.

In fixed liver sections, focal hepatocyte necrosis was found whether the animals were infected by intravenous sporozoite inoculation or by mosquito bite. Starting a few hours after sporozoite infection, mouse livers contained individual or small clusters of hepatocytes that exhibited signs of necrosis, from hydropic swelling to cell death and complete cell disintegration (Figure 7). At 3 h after intravenous inoculation of 5 × 10^6^ P. berghei sporozoites (Figure 7A) and more pronounced at 4 h and 6 h after inoculation with 5 × 10^6^ P. yoelii sporozoites (Figure 7B and 7C), we found individual or small groups of hepatocytes exhibiting various stages of necrotic cell death that were in intimate contact with neutrophil granulocytes and mononuclear inflammatory cells. Electron microscopic examination of a mouse liver 6 h after infection by bite of approximately 50 P. yoelii–infected mosquitoes showed occasional sporozoites surrounded by a parasitophorous vacuole membrane inside morphologically intact hepatocytes (Figure 8A), with neighboring hepatocytes clearly undergoing necrosis (Figure 8B and 8C). Electron microscopy failed to reveal evidence of apoptosis in any of the various liver cell types. The appearance of necrotic hepatocytes was strictly dependent on sporozoite infection; there was no parenchymal cell damage in control livers from animals exposed to uninfected mosquito bites (data not shown). The same general histopathological pattern was observed in mouse livers infected with P. berghei or P. yoelii and also in P. berghei–infected rat livers. At 40 h after inoculation of 5 × 10^6^ P. yoelii sporozoites, shortly before the release of merozoites, we found that inflammatory cells had infiltrated the mouse livers in a focal fashion; this was more pronounced at 52 h (see Figure 7D and 7E). Maturing EEFs, however, were always clear of any inflammatory reaction (see Figure 7D).

**Figure 9:**
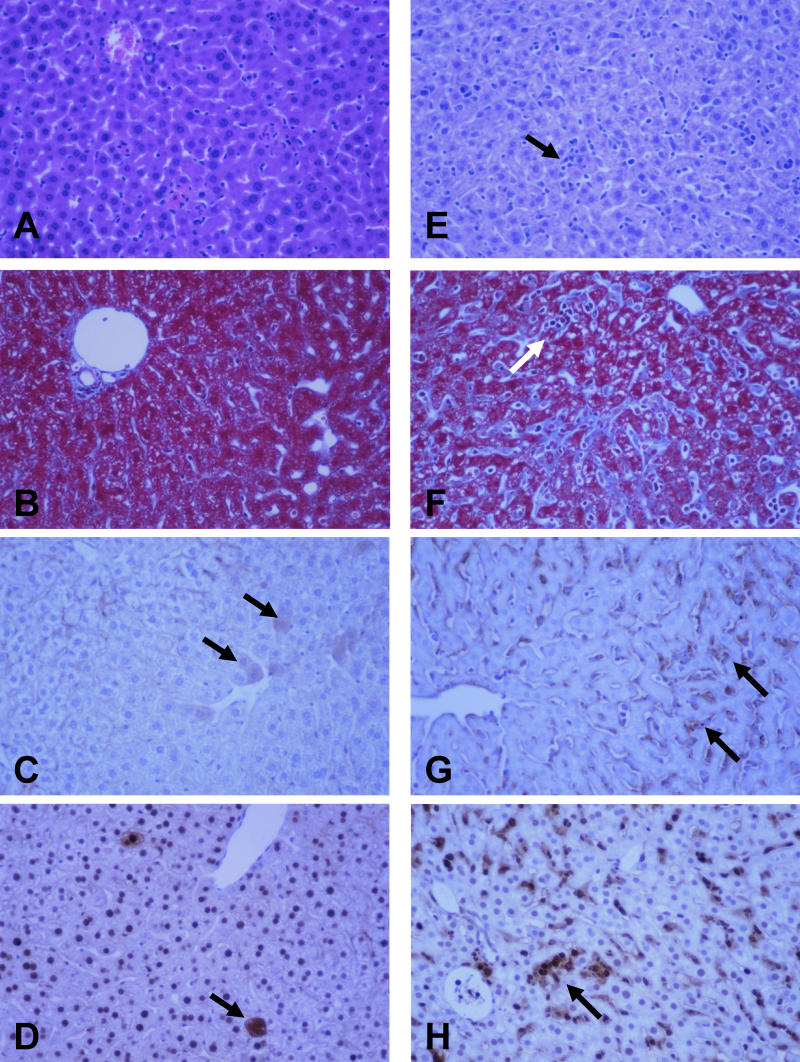
Sporozoite Transmigration Causes Histopathological Changes in the Liver Mouse livers were removed 2 d (A–D) or 7 d (E–H) after daily infection with P. yoelii by bite of 150 mosquitoes and stained with H&E (A and E) or Masson's trichrome (B and F). Other sections were subjected to immunohistochemistry using mAb PC10 against proliferating cell nuclear antigen (C and G) or mAb HHF35 against smooth muscle actin (D and H). In contrast to the livers fixed after 2 d of infection, in which only a few cells reacted with mAb PC10 and mAb HHF35 (arrows in C and D), livers examined after 7 d of infection showed (E) increased numbers of nonparenchymal cells lining the sinusoids (arrow), (F) a focal deposition of collagen (blue) in some spaces of Disse, (G) large numbers of proliferating nonparenchymal cells and hepatocytes (brown, arrows), and (H) a focal increase in the concentration of smooth muscle actin (brown, arrow).

Compared with uninfected control livers (not shown), livers from mice that had been exposed on two consecutive days to the bite of approximately 150 P. yoelii–infected mosquitoes and fixed 2 h after the second infection showed only minor alterations (Figure 9A–9D). More dramatic histopathological changes became apparent at later stages of the liver infection. This was found by infecting mice each day for a week by bite of approximately 150 P. yoelii–infected mosquitoes per day. Proliferation of blood stages was prevented by quinine treatment. Hematoxylin-eosin (H&E)–stained liver sections of these mice showed large numbers of nonparenchymal cells lining the sinusoids 7 d after infection (Figure 9E). Masson's trichrome staining revealed focal deposition of collagen in some spaces of Disse (Figure 9F). Immunohistochemistry using monoclonal antibody (mAb) PC10, directed against proliferating cell nuclear antigen, revealed hepatocyte proliferation and marked Kupffer cell hyperplasia (Figure 9G). An increased deposition of alpha-smooth muscle actin in the space of Disse (Figure 9H) was found after labeling with mAb HHF35, suggesting focal stellate cell activation [33].

### Liver Transaminases

**Figure 10:**
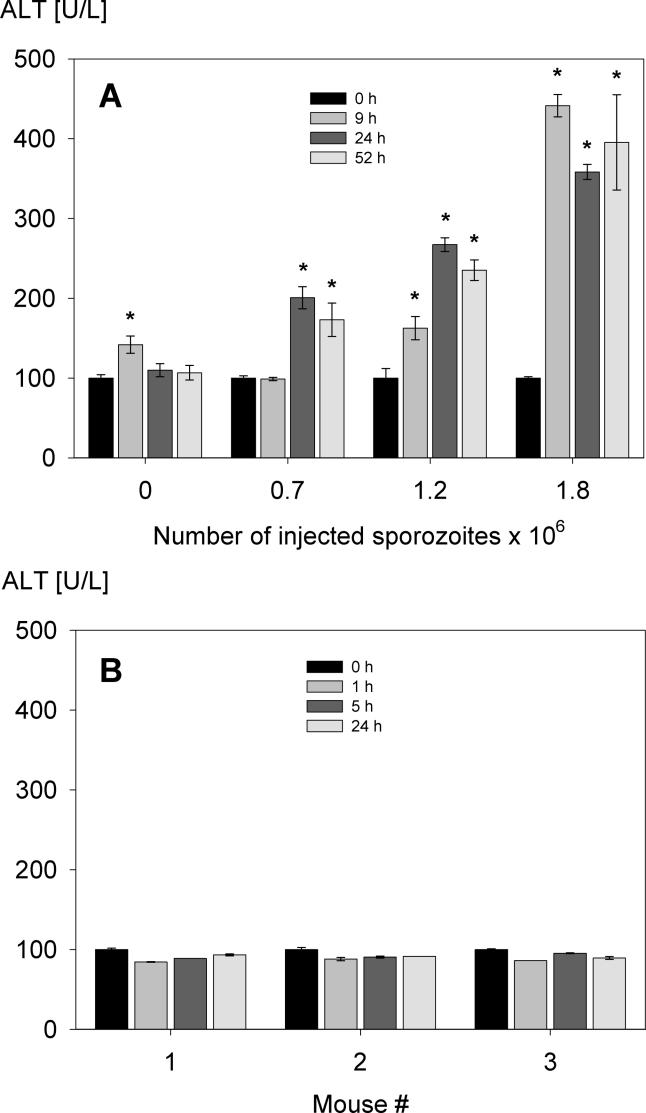
Sporozoite Infection Increases the Serum ALT Activity (A) Three mice were inoculated with salivary gland extract from 100 uninfected mosquitoes each (labeled “0” on the *x*-axis). Another three mice were infected by intravenous inoculation into the tail vein of 0.7 × 10^6^, 1.2 × 10^6^, or 1.8 × 10^6^ purified P. yoelii sporozoites (indicated as “0.7,” “1.2,” and “1.8” on the *x-*axis). The ALT activity in the serum was determined before and after infection at the indicated time points. In comparison to the control serum drawn before infection, the ALT levels increased significantly and depended on the number of inoculated sporozoites in all mice during the observation period of 52 h. Uninfected salivary gland extract had only a temporary effect on the serum ALT level (9 h). The indicated values represent the average ± standard deviation of triplicate measurements. *, *p* < 0.005 in relation to the corresponding control sera. (B) No change in the serum ALT activity was detectable when three mice were infected with P. berghei by bite of 150 mosquitoes. The indicated values represent the average ± standard deviation of duplicate measurements. For ready comparison, the data were normalized for each animal prior to statistical analysis.

Alanine aminotransferase (ALT) is commonly used as a serum marker of liver damage, and we used this to detect hepatic injury after sporozoite infection. Intravenous inoculation into mice of 0.7, 1.2, or 1.8 × 10^6^ P. yoelii sporozoites resulted in a significant increase of the ALT serum levels compared with the control sera collected before infection (Figure 10A). The degree of the increase depended on the number of injected sporozoites, and the elevated levels persisted over the entire observation period of 52 h. Because the sporozoite preparations contained debris from 60, 90, and 150 salivary glands, respectively, we also evaluated the effect of mosquito debris on the ALT activity. Extracts from uninfected salivary glands were prepared in an identical fashion, and three mice were injected intravenously with extract from 100 salivary glands each. The serum ALT levels increased temporarily at 9 h, but returned to base levels at 24 h and remained low until 52 h after inoculation (Figure 10A). The extent of this temporary increase resembled that observed after inoculation of 1.2 × 10^6^ sporozoites, which were purified from 90 salivary glands. Debris from 60 glands (containing 0.7 × 10^6^ sporozoites) had no effect at 9 h, while extract from 150 glands (1.8 × 10^6^ sporozoites) resulted in a strong elevation of the ALT levels at 9 h. Mice infected with P. berghei (Figure 10B) or P. yoelii (data not shown) by a single exposure to the bite of about 150 mosquitoes per animal did not exhibit any measurable increase in the ALT activity in the serum.

## Discussion

**Figure 11:**
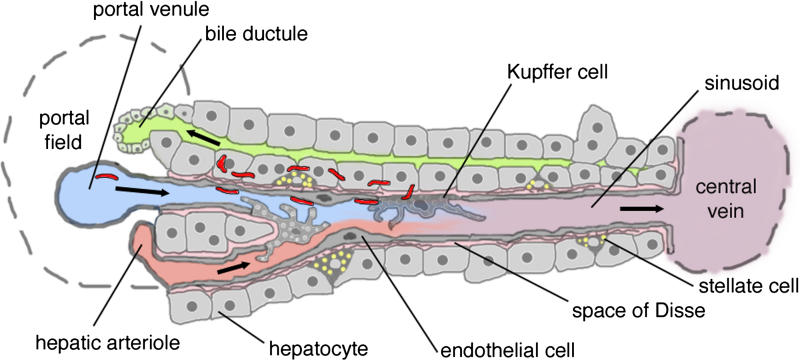
Model of *Plasmodium* Sporozoite Infection of the Mammalian Liver The dual blood supply of the liver, consisting of branches of the portal vein and the hepatic artery, merges upon entry into the liver lobule at the portal field. The blood flows along the sinusoid and exits at the central vein. First sporozoites enter the liver lobule either via the portal vein or the hepatic artery, and then are abruptly arrested by binding to the sinusoidal cell layer. The initial binding is presumably mediated by stellate-cell-derived ECM proteoglycans that protrude from the space of Disse across the endothelial sieve plates into the sinusoidal lumen. After a pause, the parasites begin to glide along the sinusoid, frequently moving against the bloodstream, until they then encounter a Kupffer cell, on the surface of which they recognize selected chondroitin and heparan sulfate proteoglycans. Sporozoites position themselves with their apical cell pole facing the phagocyte. After a considerable pause, they slowly pass through the Kupffer cell and cross the space of Disse beyond it, exhibiting a clearly visible constriction. Once inside the liver parenchyma, the parasites increase their velocity and migrate for many minutes through several hepatocytes, before they eventually settle down in a final one for EEF development. Sporozoite transmigration results in a trail of necrotic hepatocytes, whose remains are subsequently removed by infiltrating inflammatory cells.

Intravital microscopy enabled us to observe and document the cascade of events leading to malaria sporozoite infection of the liver. Our recordings show that sporozoites entering the liver lobule are abruptly arrested by binding to the sinusoidal cell layer, presumably by recognizing liver-specific ECM proteoglycans protruding through the endothelial fenestration into the sinusoidal lumen [4]. Sporozoites then glide along the sinusoid until they encounter a Kupffer cell, where they stop with their apical end attached to the phagocyte. After a pause, they push slowly across the sinusoidal cell barrier, showing a point of constriction in their outline. Once inside the liver parenchyma, they continue to migrate for many minutes, fatally wounding several hepatocytes in the process. Eventually, the parasites settle down in one final hepatocyte, become surrounded by a parasitophorous vacuole membrane, and differentiate to EEFs (model in Figure 11).

The overall velocities of sinusoidal gliding and hepatocyte transmigration are similar to sporozoite gliding in Matrigel (approximately 1.8 μm/s; see Table 1), on artificial surfaces in vitro (1–3 μm/s) [34], and in mosquito salivary glands (≤ 2 μm/s) [27], but migration through Kupffer cells is slower by an order of magnitude. The speeds of sinusoidal gliding and hepatocyte transmigration (Table 2) fall into the general speed range of 1–10 μm/s found for various other apicomplexan parasites [35,36], including Toxoplasma gondii tachyzoites [37], Eimeria tenella sporozoites [38], and Gregarina polymorpha [35]. Interestingly, the velocity of *Plasmodium* ookinetes (0.08–0.25 μm/s) is slower than that of sporozoites [39,40] and similar to that of sporozoites traversing Kupffer cells, perhaps reflecting an adaptation to the environment of the mosquito midgut.

**Table 2:**
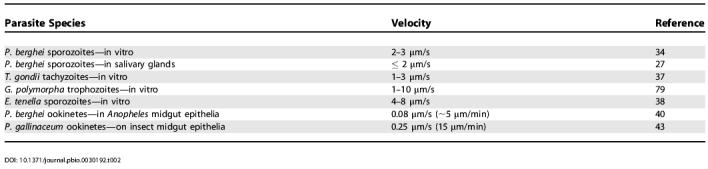
Gliding Speeds of Apicomplexan Parasites

The process of Kupffer cell passage differs from passage through hepatocytes by more than speed. Entry into hepatocytes does not involve a pause before entry, a constriction in the parasite during passage, or a change in migration speed (see Table 1). Perhaps the slower speed relates to the need to form a vacuole or duct for passage through Kupffer cells [14,15,41] in contrast to direct breaching of the cell membrane during transmigration through hepatocytes [25,42]. Alternatively, the barrier that causes the constriction and slows the parasites could be the ECM in the space of Disse just beyond the Kupffer cell. A similar phenomenon has been observed for P. gallinaceum ookinetes, which cross the microvilli-associated network of Aedes aegypti midgut epithelia at roughly 10-fold slower a speed compared with the subsequent migration on or through the cells [40,43]. However, since host cell entry can also cause the formation of a constriction in apicomplexan parasites [44−46], additional work is required to identify the nature of the barrier presented to sporozoites leaving the sinusoid.

In contrast to motility on artificial surfaces in vitro, where sporozoites maintain a fixed crescent shape and move in circles or spirals [34,47], sporozoite migration in vivo and in Matrigel is characterized by a more linear migration pattern, high parasite flexibility, and frequent changes in direction, most likely guided by structural tissue components. The general movement pattern in the liver resembles that of sporozoites gliding in the skin after transmission by mosquito bite [1]. P. berghei sporozoites in *An. stephensi* salivary glands also move according to tissue architecture and follow the outline of the acinar epithelia and the secretory duct [27]. It appears that *Plasmodium* sporozoites, in general, are guided by the three-dimensional arrangement of matrix structures to reach their next destination in a tissue.

We were surprised to find that P. berghei sporozoites traverse a similar number of hepatocytes in livers from mice and rats. P. berghei is much less infectious to mice than to its natural host, the African wild rat Thamnomys surdaster [48,49], and induces a more pronounced inflammatory reaction in these rodents, while young rats exhibit intermediate levels of infection and inflammation [50–52]. Based on our data, it appears that sporozoite transmigration is an intrinsic feature of *Plasmodium* sporozoites, which occurs irrespective of the host–parasite combination. Thus, it may be the degree of the mismatch between parasite and host that determines the extent of the inflammatory response to hepatocyte necrosis.

Malaria sporozoite migration can cause significant injury to the liver tissue, as demonstrated by the inoculation-size-dependent increase in the serum ALT levels for at least 2 d after intravenous inoculation of P. yoelii sporozoites (see Video S12). Uninfected gland extracts had a significant, albeit temporary, effect, suggesting that the sustained ALT elevation observed 24 and 52 h after sporozoite inoculation was indeed a consequence of parasite migration. Although the ALT levels measured here are well below those observed in experimental fulminant hepatitis caused by murine hepatitis virus 3 infection [53], the sporozoite-induced damage was large enough to induce deposition of detectable amounts of collagen in the space of Disse, focal Kupffer cell hyperplasia, and groups of proliferating hepatocytes at later time points. The liver damage induced by bite of infected mosquitoes, although morphologically detectable, remained below the detection limit of the ALT assay, most likely because the number of sporozoites reaching the liver under natural transmission conditions is too low to induce a significant hepatotoxic effect. Mosquitoes generally transmit less than 100 sporozoites per bite [1,50,54,55], so under optimal conditions 150 mosquitoes would have transmitted roughly 15,000 sporozoites per mouse, only part of which leave the skin and travel to the liver (J. Vanderberg and U. Frevert, unpublished data). These findings also suggest either that mosquitoes do not inject significant amounts of saliva into the bloodstream while probing the skin or that saliva has no adverse effect on the liver parenchyma. In conclusion, while experimental infection with large numbers of purified sporozoites can inflict measurable liver injury, natural infection by mosquito bite poses no risk to liver function, even when small rodents are exposed to large numbers of well-infected mosquitoes under laboratory conditions. These data confirm that sporozoite-induced liver injury is of no concern to malaria-infected individuals living in endemic areas.

A contradictory model of sporozoite entry into the liver was recently presented by Yuda and colleagues [23]. The group generated P. berghei parasites deficient in the micronemal proteins SPECT1 and SPECT2, which exhibit greatly diminished liver infectivity after intravenous inoculation into mice. Experimental data on the function of the SPECT proteins are lacking to date, but SPECT2 has been proposed to contain a membrane attack complex that mediates membrane wounding and, consequently, sporozoite transmigration through cells. In vitro results obtained from permanent nonphagocytic cell lines supported this hypothesis, but the authors then extrapolated from these data to Kupffer cells, hypothesizing that SPECT mutants have lost their ability to wound the Kupffer cell membrane and therefore cannot pass through the cytoplasm of these phagocytes. This is in contrast to Kupffer cell passage by membrane invagination as documented by others [14,15,41]. To explain this discrepancy and taking into account the putative membrane attack complex in SPECT2, one could speculate that SPECT mutants accumulate inside Kupffer cells because they have lost the ability to escape the vacuole surrounding them in these cells.

In another study by Ishino and colleagues, Kupffer cell elimination more than doubled the rate of liver infection by P. berghei [24]; this would not be possible at the approximately 80% infection rate presented here. The most likely explanation for this discrepancy is that our results are based on natural sporozoite transmission by mosquito bite, while Ishino and colleagues used intravenous injection, which results in markedly lower liver infection efficiencies relative to the size of the inoculum [56]. It is generally believed that preparations of purified sporozoites contain a considerable percentage of noninfectious parasites, the majority of which is likely cleared from the bloodstream by Kupffer cells. Kupffer cells may become activated upon phagocytosis of dead sporozoites, and the resulting inflammatory microenvironment would clearly inhibit EEF development [57]. This would not occur in the absence of Kupffer cells [58], thus explaining the large increase in the liver infection rate in clodronate-treated mice. In addition, clodronate released by leakage from liposomes or by dying Kupffer cells has been suggested to suppress inflammatory cytokines [58], which would also enhance EEF survival**.**

*Plasmodium* sporozoites continuously release vesicles covered with CSP and thrombospondin-related adhesive protein from their cell surface membrane [47,59–61]. When gliding on artificial surfaces such as glass, sporozoites leave these vesicles behind in the shape of a trail, but when migrating on cultured cells or in tissues such as mosquito salivary glands, the released CSP translocates across membranes; it initially spreads across the cytoplasm of the affected cells and inhibits protein synthesis and later redistributes to the perinuclear space [62,63]. Since sporozoites release CSP into Kupffer cells in vitro [14], we expect that this also occurs with liver endothelia and Kupffer cells in vivo. The significance of this is that both sinusoidal endothelia and Kupffer cells are fully mature antigen-presenting cells that express major histocompatibility complex class I and II molecules as well as the co-stimulatory molecules CD80 and CD86, they are able to prime naïve CD4^+^ T cells in vitro, and they can cross-present alloantigen to CD8^+^ T cells [64–67]. Intimate contact between sporozoites and nonparenchymal cells could be expected to lead to the presentation of parasite antigen—in particular, CSP-derived peptides. However, due to its ribotoxic effect, translocated CSP may interfere with antigen processing. In addition, the unique microenvironment of the liver generally favors the development of tolerance rather than inflammation and immunity, a property thought to have evolved to avoid overreaction to the continuous influx into the portal circulation of foreign materials such as bacteria and endotoxins from the intestines [68,69]. Portal vein tolerance [70] is predominantly mediated by Kupffer cells [71], can occur irrespective of nature and origin of the antigen and has been implicated in the acceptance of liver allografts, the reduced rejection of allografts when preceded by portal venous administration of donor antigen, the high frequency of tumor metastases in the liver, and the persistence of intrahepatic pathogens such as hepatitis C and hepatitis B virus [72,73]. Does this unusual route of entry, together with translocation of CSP by migrating sporozoites, enable malaria sporozoites to take advantage of the unique tolerogenic nature of the liver? Can *Plasmodium,* one of the deadliest infectious agents worldwide, avoid the generation of an effective immune response against the continuous influx of sporozoites in endemic areas so successfully because it begins its life cycle in the mammalian host with a round of multiplication in the liver? The liver may be the preferred site for multiplication because of its unique nature as an immune organ as opposed to other tissues that could have possibly supported replication but do not possess this specialized property.

## Materials and Methods

### 

#### Parasites.

*An. stephensi* mosquitoes were used to propagate (1) P. berghei (NK65), (2) P. yoelii (17 XNL), or (3) a P. berghei clone that expresses EGFP at the sporozoite stage under control of the CSP promoter (termed here GFP P. berghei) [74]. Another P. berghei clone was generated that expresses an improved version of the red fluorescent protein drFP583/DsRed/RFP, RedStar, under the control of the CSP promoter (termed here RedStar P. berghei); RedStar was chosen because of its 10–20× enhanced brightness when expressed in mammalian cells [75]. The transfection vector pSE-26 contains a RedStar expression cassette and is a derivative of the b3Djournal-pbio-0030192-transfection vector [76] that confers resistance to pyrimethamine. The RedStar open reading frame was amplified from the plasmid PRS415-Gal1-RedStar [75] (a gift from M. Knop, Heidelberg) with primers RFPfor (5′ CGGGATCCAAAATGAGTAGATCTTCTAAGAAC 3′; BamHI site is underlined) and RFPrev (5′ GGACTAGTTTACAAGAACAAGTGGTGTCTACC 3′; SpeI site is underlined). The 3′ UTR region of *PbDHFR* was amplified from pExpEF (a gift from A. P. Waters, Leiden) with primers SEb3DforXbaI (5′ TGCTCTAGACGTTTTTCTTACTTATATAT 3′; XbaI site is underlined) and SEb3CrevSacII (5′ TCCCCGCGGCGGTGTGAAATACCGCACAGA 3′; SacII site is underlined). To drive stage-specific expression, the P. berghei CSP promoter was amplified using primers p5′CSEcoRIfor (5′ CCGGAATTCACATAAAAGGGAATATGGAATATACTAGC 3′; EcoRI site is underlined) and p5′CSBamHIrev (5′ CGCGGATCCAAATATATGCGTGTATATATAGATTTTG 3′; BamHI site is underlined) and genomic PbDNA. Parasite transfection and selection was performed as described previously [76]. Serial dilution of the parental pyrimethamine-resistant parasites resulted in three independent clonal parasite lines that stably express RedStar during sporozoite stages.

#### Animals

Mice were (1) Balb/c (Taconic, Germantown, New York, United States); (2) Swiss Webster (Taconic); (3) a transgenic strain expressing GFP in vascular endothelial cells under control of the Tie2 promoter [29]; (4) lys-EGFP-ki mice that express EGFP in myelomonocytic cells—in particular, neutrophil granulocytes—and tissue macrophages including Kupffer cells [30]; or (5) Tie2-GFP × lys-EGFP-ki hybrid mice. The lys-EGFP-ki mice were a kind gift from Dr. Thomas Graf, Albert Einstein College of Medicine, Bronx, New York. Brown Norway rats were purchased from Charles River (Wilmington, Massachusetts, United States).

#### Surgery and intravital microscopy

Mice were anesthetized by intraperitoneal injection of a cocktail of 50 mg/kg of ketamine (Ketaset, Fort Dodge Animal Health, Overland Park, Kansas, United States), 10 mg/kg of xylazine (AnaSed, Ben Venue Laboratories, Bedford, Ohio), and 1.7 mg/kg of acepromazine (Boehringer Ingelheim Vetmedica, St. Joseph, Missouri, United States). A portion of the right abdominal skin was removed and the peritoneal cavity opened along the rib cage. The mouse was then placed on the stage of an inverted Nikon (Tokyo, Japan) Diaphot microscope equipped with a Cooke SensiCam digital camera (Cooke, Romulus, Michigan, United States). The liver was immobilized with gauze and kept moist with warm PBS. Sporozoite infection was done on the microscope stage by bite of 100−200 infected mosquitoes. Images of sporozoites entering the liver were captured using either a fluorescein isothiocyanate long-pass filter combination or a GFP/DsRed dual band filter set (Chroma Technology, Rockingham, Vermont, United States) and imported into Image-Pro Plus software (Media Cybernetics, Silver Spring, Maryland, United States). Typical exposures times were 100 ms per image for gray tone and 300 ms for RGB images. Phototoxicity was limited by reducing light transmission to 20% with a neutral density filter.

#### Infection of mouse livers for histopathology.

Mice were intravenously inoculated with 2–5 × 10^6^ purified P. berghei or P. yoelii salivary gland sporozoites, and the livers were removed 0.5, 1, 2, 4, 6, 12, 24, or 48 h after infection. Other mice were infected with P. berghei or P. yoelii by bite of 100–200 mosquitoes. For observation periods longer than 2 d, blood infection was prevented by daily administration of 70 mg/kg of quinine hydrochloride (Sigma, St. Louis, Missouri, United States). The tissue was fixed 2 h after the last infection with PBS containing 4% paraformaldehyde and with PBS containing 4% paraformaldehyde plus 1% glutaraldehyde for confocal and electron microscopic examination, respectively [5].

#### Immunofluorescence microscopy

Ten-micrometer frozen sections were prepared with a Reichert-Jung (Arnsberg, Germany) Frigocut cryostat, and 1-μm cryosections were cut with an RMC (Tucson, Arizona, United States) MT-7 ultramicrotome equipped with a MRC-21 cryochamber. Sporozoites were labeled with mAb 3D11 [77] or mAb NYS1 [78] directed against the P. berghei and P. yoelii CSP, respectively, followed by protein A conjugated to fluorescein isothiocyanate. The liver tissue was counterstained with 0.1% of Evans blue in PBS.

#### Confocal microscopy

Immunofluorescence-labeled frozen liver sections were examined with a Zeiss (Oberkochen, Germany) LSM 510 laser scanning microscope. Whole unfixed livers were examined for GFP-expressing parasites and liver cells by scanning through the intact capsule of the organ. The natural autofluorescence of the tissue was excited at 543 nm and recorded at 560–615 nm to visualize the microarchitecture of the organ.

#### Histopathology and immunohistochemistry

Paraffin-embedded liver tissue was processed for H&E and Masson's trichrome staining. Immunohistochemistry was performed on deparaffinized mouse liver sections using antibody PC10 against proliferating cell nuclear antigen and antibody HHF35 against muscle-specific actin as a marker specific for dedifferentiating stellate cells (both from Ventana Medical Systems, Tucson, Arizona, United States).

#### Electron microscopy

Mouse liver tissue was postfixed with 1% osmium tetroxide, dehydrated, and embedded in Epon as described [62]. Thin sections were cut with an RMC MT-7 ultramicrotome and post-stained with uranyl acetate and lead citrate. Photographs were taken with a Zeiss EM 910 electron microscope.

#### Image processing

Electron microscopy negatives were scanned with an Agfa (Mortzel, Belgium) Horizon Plus flatbed scanner. Digital, electron, or confocal microscopy images were processed using Image-Pro Plus, Adobe Photoshop (Adobe Systems, San Jose, California, United States), AutoDeBlur (AutoQuant Imaging, Troy, New York, United States), and Microsoft (Redmond, Washington, United States) PowerPoint software.

#### Liver transaminases.

Mice were anesthetized and infected with P. berghei or P. yoelii by bite of 150 mosquitoes per animal. *An. stephensi* mosquitoes were allowed to probe the skin of the mice for 5 × 2 min. Blood was drawn from the tail vein before and at various time points for up to 52 h after infection. Other mice were infected by inoculation into the tail vein of 0.7 × 10^6^, 1.2 × 10^6^, and 1.8 × 10^6^ P. yoelii sporozoites, which were purified from 60, 90, and 150 salivary glands, respectively. Control mice were intravenously injected with identically prepared extracts from 100 uninfected salivary glands each. The serum ALT levels were measured at the indicated time points using a GPT/ALT assay (Wako Chemicals, Richmond, Virginia, United States) according to manufacturer's instructions. The values represent the average of triplicate measurements ± standard deviation.

## Supporting Information

Digital movies document the individual steps of the P. berghei sporozoite infection cascade of the mouse or rat liver. Replay speed = 50×.

Video S1 Intravital Microscopy of the Sinusoidal Blood Flow in a Tie2-GFP Mouse Liver Note the dark blood cells traveling inside the sinusoids, which are lined with GFP-expressing endothelia. GFP is particularly prominent in the perinuclear region of the endothelia.
(656 KB ZIP).

Video S2 Intravital Microscopy of the Sinusoidal Blood Flow in a lys-EGFP-ki Mouse Liver Bright green fluorescent blood granulocytes are traveling rapidly inside the sinusoids. Kupffer cells are stationary and can be identified by their star-like shape and their lower fluorescence signal.
(656 KB ZIP).

Video S3 Intravital Microscopy of RedStar Sporozoite Gliding along Liver Sinusoids P. berghei sporozoites entering the liver lobule are abruptly arrested by binding to the sinusoidal endothelium. After a brief pause, the parasites begin to glide along the sinusoidal cell layer. Periodically, the parasites lose their grip to the sinusoidal cell layer and are swept with the bloodstream for a short distance before they adhere again and continue to glide. This movie clip shows a RedStar P. berghei sporozoite gliding with or against the bloodstream in a Tie2-GFP mouse liver.
(1.5 MB ZIP).

Video S4 Intravital Microscopy of GFP Sporozoite Gliding along Liver Sinusoids This movie clip shows a GFP P. berghei sporozoite gliding with or against the bloodstream in a Tie2-GFP mouse liver.
(1.1 MB ZIP).

Video S5 Intravital Microscopy of Sporozoites Passing through Kupffer Cells After encountering a Kupffer cell in a lys-EGFP-ki mouse liver, a GFP P. berghei sporozoite pauses for 1–2 min, facing the phagocyte with its apical cell pole. It then passes through the Kupffer cell at a slow speed, pushing through a narrow constriction. Once inside the liver parenchyma, the sporozoite increases its speed and continues its migration through several hepatocytes.
(2.6 MB ZIP).

Video S6 Intravital Microscopy of a Kupffer Cell Passage Another sporozoite glides inside a sinusoid before it encounters a Kupffer cell in a lys-EGFP-ki mouse liver. After a pause, it traverses the phagocyte and continues its migration in the liver tissue for many minutes.
(2.1 MB ZIP).

Video S7 Sporozoite Transmigration through Hepatocytes Occurs in Mouse and Rat Livers Typical transmigration behavior of GFP P. berghei sporozoites in a Tie2-GFP mouse liver.
(907 KB ZIP).

Video S8 Sporozoites Migrate through Several Hepatocytes in Mouse and Rat Livers Typical transmigration behavior of GFP P. berghei sporozoites in the livers of young Brown Norway rats.
(1.2 MB ZIP).

Video S9 All Sporozoites That Adhere to the Sinusoidal Cell Layer Do Not Enter the Liver The GFP P. berghei sporozoite does not bind tightly to the surface of the sinusoidal cell layer. It retains a fixed crescent shape and appears paralyzed or dead. Eventually, the parasite is displaced from the sinusoidal cell layer by the shear force of the bloodstream and flushed out of the liver lobule.
(2.5 MB ZIP).

Video S10 Sporozoites Reentering a Sinusoid Are Swept Away with the Bloodstream This movie clip shows a GFP P. berghei sporozoite that has successfully entered the liver tissue and is in the process of transmigrating in the liver parenchyma. However, the parasite accidentally reenters a sinusoid and is flushed out of the liver lobule.
(893 KB ZIP).

Video S11 Intracellular Sporozoite Migration Affects the Subcellular Structure This confocal microscopic movie clip shows a GFP P. berghei sporozoite circling around the nucleus of a CHO-K1 cell in vitro. The clearly visible displacement of cell organelles by the parasite suggests that transmigrating sporozoites can disrupt vital functions in hepatocytes as a consequence of the destruction of the cellular architecture—in particular, the abundant, well-developed rough endoplasmic reticulum.
(839 KB ZIP).

Video S12 Sporozoite Passage across Tissue Barriers Occasionally, GFP P. berghei sporozoites can be observed crossing unidentified tissue barriers by propelling themselves forward in a manner that cannot solely be explained by gliding motility.
(662 KB ZIP).

Video S13 Sporozoite Migration in Matrigel The sporozoite migration pattern in Matrigel resembles that in the liver or the skin. GFP P. berghei sporozoites were purified from salivary glands, suspended in Matrigel, and incubated at 37 °C for 30 min before recording.
(149 KB ZIP).

### Accession Numbers

The GenBank (http://www.ncbi.nlm.nih.gov/Genbank/) accession number for red fluorescent protein drFP583/DsRed/RFP is AAF03369.

## Abbreviations

ALT: alanine aminotransferase
CSP: circumsporozoite protein
ECM: extracellular matrix
EEF: exoerythrocytic form
GFP: green fluorescent protein
H&E: hematoxylin-eosin
mAb: monoclonal antibody;
